# Effect of Size and Concentration of PLGA-PEG Nanoparticles on Activation and Aggregation of Washed Human Platelets

**DOI:** 10.3390/pharmaceutics11100514

**Published:** 2019-10-04

**Authors:** Rana Bakhaidar, Joshua Green, Khaled Alfahad, Shazia Samanani, Nabeehah Moollan, Sarah O’Neill, Zebunnissa Ramtoola

**Affiliations:** 1 School of Pharmacy and Biological Sciences, Royal College of Surgeons in Ireland, 2 Dublin, Ireland; ranabakhaidar@rcsi.ie (R.B.); soneill@rcsi.ie (S.O.); 2 School of Medicine, Royal College of Surgeons in Ireland, 2 Dublin, Ireland; joshuagreen@alumnircsi.com (J.G.); khaledalfahad@rcsi.ie (K.A.); shaziasamanani@rcsi.ie (S.S.); nabeehahmoollan@alumnircsi.com (N.M.)

**Keywords:** PLGA-PEG, nanoparticles, platelet, activation, aggregation, binding, uptake

## Abstract

Nanotechnology is being increasingly utilised in medicine as diagnostics and for drug delivery and targeting. The small size and high surface area of nanoparticles (NPs), desirable properties that allow them to cross biological barriers, also offer potential for interaction with other cells and blood constituents, presenting possible safety risks. While NPs investigated are predominantly based on the biodegradable, biocompatible, and FDA approved poly-lactide-*co*-glycolide (PLGA) polymers, pro-aggregatory and antiplatelet effects have been reported for certain NPs. The potential for toxicity of PLGA based NPs remains to be examined. The aims of this study were to determine the impact of size-selected PLGA-PEG (PLGA-polyethylene glycol) NPs on platelet activation and aggregation. PLGA-PEG NPs of three average sizes of 112, 348, and 576 nm were formulated and their effect at concentrations of 0.0–2.2 mg/mL on the activation and aggregation of washed human platelets (WP) was examined. The results of this study show, for the first time, NPs of all sizes associated with the surface of platelets, with >50% binding, leading to possible internalisation. The NP-platelet interaction, however, did not lead to platelet aggregation nor inhibited aggregation of platelets induced by thrombin. The outcome of this study is promising, suggesting that these NPs could be potential carriers for targeted drug delivery to platelets.

## 1. Introduction

Biocompatible and biodegradable nanoparticles (NPs) continue to be extensively investigated for the diagnosis, prevention, and therapy of various diseases, for cellular and molecular imaging, and for tissue engineering applications [1,2,3,4,5]. NPs have been reported to target various therapeutic agents to the central nervous system (CNS), tumour sites, and the vascular compartment [6,7,8,9,10]. The small size and high surface area of NPs are desirable properties that allow them to cross biological barriers, but also offer potential for interaction and interference with other cells and blood constituents, presenting possible safety risks. While the NPs investigated are predominantly based on the biodegradable, biocompatible and FDA approved poly-lactide-*co*-glycolide (PLGA) polymers, irrespective of their intended destination, NPs introduced into the bloodstream will interact with endothelial cells as well as blood constituents. It is therefore important to understand the bio-distribution of such NPs in human blood and their interaction with various blood components such as platelets [6,11,12]. Previous studies report that grafting a polyethylene glycol (PEG) chain to a PLGA polymer backbone significantly increases the circulation half-life of the modified copolymer, as it remains camouflaged from the macrophage [13,14]. The longer duration of exposure to blood components can result in enhanced interaction potential with blood cells, and in particular with platelets, maximising the possibility of modulating platelet, activation, secretion, and aggregation [15,16].

NP physicochemical characteristics, such as size and net charge (zeta potential (ZP)) that are reported to be key factors enhancing their efficacy, can substantially affect their bio-distribution [17,18]. Variations in NP characteristics may result in a diverse range of platelet reactivity but unfortunately, only a few studies regarding the interaction of NPs with washed platelets (WPs) have been published [18,19,20]. We previously reported that biodegradable micro and NPs of PLGA, PLGA-PEG, and chitosan, of median diameter (D50%) of 2–9 µm and 100–500 nm, and with various surface morphology did not induce or inhibit platelet aggregation at particle concentrations of 0.1–500 µg/mL [20]. In contrast, silica NPs of 10 nm, 50 nm, and 150 nm, were shown to cause platelet hyper-aggregability, promoting the risk of thrombus deposition, while particles of 500 nm did not pose such a risk [21]. Mixed carbon NPs, carbon nanotubes, single-walled nanotubes, and multi-walled nanotubes were reported to stimulate platelet aggregation by the upregulation of GPIIb/IIIa receptors on platelets, inducing subsequent vascular thrombosis [19]. In a separate study, the contribution of silver NPs to the effective inhibition of integrin-mediated WPs responses, such as aggregation, secretion, and adhesion to immobilized fibrinogen or collagen, was established [22].

The pro-aggregatory effects of some NPs and the antiplatelet effects of others raise questions regarding the safety of biodegradable nanomaterials in terms of interference with the haemostatic equilibrium [20,23]. To date, the potential interactions of PLGA-PEG NPs with blood elements remains scant. In particular, the effect of their size on blood constituents, as well as any potential hazard they may pose, have not been studied. Platelet activation is a precisely regulated event, critical for maintaining normal blood flow. When designing drug-loaded NPs intended to be delivered to the systemic circulation, a major consideration is to maintain platelets in an inactive state [22].

The aims of this study were to determine the impact of size-selected PLGA-PEG NPs on platelet activation and aggregation. Coumarin-6-labelled PLGA-PEG NPs of three average sizes of 112, 348, and 576 nm were formulated using the solvent dispersion technique [20]. The effect of NP size and NP concentration of 0.01–2.2 mg/mL on the activation and aggregation profiles of washed platelets (WP) were examined using platelet aggregation assays and flow cytometry. Confocal imaging was carried out on NPs incubated with WP to characterise the interaction of NPs with platelets in the resting and activated states.

## 2. Materials and Methods

### 2.1. Materials

Albumin, from human serum, Coumarin-6 (98%), Polysorbate 80 (Tween^®^ 80), Dextrose, Sodium Chloride (NaCl), Sodium bicarbonate (NaHCO_3_), Potassium Chloride (KCl), Sodium Citrate (Tribasic, 10 dehydrate (HOC(COONa)(CH_2_COONa)_2_, KH_2_PO_4_ (monobasic potassium phosphate anhydrate or potassium dihydrogen phosphate), Calcium Chloride (CaCl_2_), Magnesium Chloride hexahydrate MgCl_2_·6H2O, and HEPES (*N*-2-Hydroxyethylpiperazine-*N*’-2-Ethanesulfonic Acid) were purchased from Sigma-Aldrich Dublin, Ireland. PLGA-PEG containing PEG at 10% *w*/*w* (poly-d, l-lactic-*co*-glycolic acid-polyethylene glycol diblock copolymer 50:50 mPEG; 33 kDa with inherent viscosity: 0.05–0.15 dL/g, Lakeshore Biomaterials™, was purchased from Evonik Industries AG, Essen, Germany. Deionised water was used throughout the experiments.

### 2.2. Methods

#### 2.2.1. Preparation and Characterisation of PLGA-PEG NPs

PLGA-PEG NPs containing a fluorescent marker, coumarin-6, were prepared using the solvent dispersion method [24]. Briefly, PLGA-PEG polymer was dissolved in acetone to form 10, 55, and 100 mg/mL solutions, and coumarin-6 at 0.05% *w*/*w* of the polymer was dissolved in the PLGA-PEG solutions. The polymer solutions were added dropwise to an external aqueous phase containing Tween 80^®^ at 2% *w*/*v*, under constant stirring. The NPs formed were recovered by centrifugation at 11,000 rpm for 20 min (Rotina 35 R centrifuge, Hettich Zentrifugen, Tuttilingen, Germany). NPs were washed with deionised water, centrifuged, and the pellet resuspended in deionised water and stored at 4 °C. The average particle size (PS), polydispersity index (PDI), and zeta potential (ZP) of the NPs were determined by dynamic light scattering technique, using a Malvern Zetasizer Nano ZS 90 (Malvern Instruments, Worcestershire, UK). After centrifugation, NPs were suspended in deionised water, placed in disposable voltable zeta cells (DTS 1060) for analysis using DLS. Viscosity and refractive index of the dispersants were taken into account. For each sample, the average of five measurements was calculated. Results were expressed as an average of five measurements ± standard deviation.

The morphologies of NPs were examined under a MIRA3 variable pressure field emission scanning electron microscope (Tescan, Brno-Kohoutovice, Czech Republic) at an accelerating voltage of 5.0 kV and magnification of 30,000 to 50,000×. Samples of lyophilised NPs were applied onto aluminium stubs using a double-sided conductive tape and were sputter-coated with gold.

#### 2.2.2. Preparation of Washed Platelets

Venous whole blood was obtained from healthy human volunteers, free from aspirin and other non-steroidal anti-inflammatory agents for the previous 7–10 days. Ethical approval was obtained from the Research Ethics Committee, Royal College of Surgeons in Ireland. The blood obtained was centrifuged at 180 g for 12 min, to separate the platelet-rich plasma (PRP) that was acidified to pH 6.5 and the plasma pelleted by centrifugation at 720 g for 12 min. Washed platelets were then adjusted to approximately 250 × 10^9^ platelets per millilitre by resuspending the pellets in HEPES (*N*-2-Hydroxyethylpiperazine-*N*’-2-Ethanesulfonic Acid) platelet buffer and leaving to stand for 30 min before adding calcium chloride (CaCl_2_) at a final concentration of 1.8 mM.

#### 2.2.3. Effect of Size and Concentration of NPs on Platelet Aggregation

Platelet aggregation was determined by measuring the change in the optical density of stirred WPs in the absence or presence of 0–2.2 mg/mL PLGA-PEG NPs of different sizes, following the addition of the platelet agonist, thrombin. Platelets were incubated with NPs for 4 min prior to the addition of 0.1 U/mL thrombin. Platelet aggregation data was analysed using an eight channel platelet aggregometer,-PAP-8 (Bio/Data Corporation, Horsham, PA, USA). Aggregation results were expressed as final percent aggregation (% PA) at the end of the reaction time of 12 min. Data are presented as mean of *n* = 4–6 ± SEM.

#### 2.2.4. Effect of Size and Concentration of NPs on Platelet Activation

WPs (250 × 10^3^/µL) were incubated with 20 µL phycoerythrin (PE)-labelled CD62P antibody and mixed with PLGA-PEG NPs of different sizes, in the absence and presence of 0.1 U/mL thrombin and incubated for 4 min at 37 °C. At predetermined time points, samples were fixed using 1% *v*/*v* formaldehyde (FA) and platelet activation was measured by flow cytometry. Platelet activation was determined by measuring the level and extent of CD62P antibody binding to the platelet surface, expressed as percent positive cells (% PP) and mean fluorescence intensity (MFI). Platelets with and without CD62P were used as negative controls and platelets activated with thrombin at 0.1 U/mL for 4 min was the positive control. Data are average of *n* > 3 ± SEM.

#### 2.2.5. Confocal Microscopy of the Effect of Incubation Time on The Interaction of PLGA-PEG NPs with Washed Platelets

Coumarin-labelled NPs of 112, 348, and 576 nm, at 0.1 and 2.2 mg/mL, were incubated with resting WPs in suspension for 1, 5, 15, and 30 min. At each time point, samples were fixed with 1% formaldehyde, stained red with phalloidin-tetramethylrhodamine isothiocyanate (phalloidin-TRIT-C) and examined using confocal laser scanning microscopy (CLSM), (Carl Zeiss, Jena, Germany). WPs were also incubated with coumarin-labelled NPs of the three different sizes at 2.2 mg/mL for 1, 5, and 30 min, and allowed to attach to fibrinogen-coated glass slides (20 µg/mL). At each time point, samples were fixed with 1% formaldehyde, platelets stained red with phalloidin-TRITC, and examined by CLSM.

#### 2.2.6. Statistical Analysis

Statistical analysis was carried out using GraphPad Prism (version 7.00 for Windows; GraphPad Software, San Diego, CA, USA). One-way ANOVA and unpaired student T tests and One-way ANOVA followed by post hoc analysis using Dunnett’s Tests were used to determine differences between samples and groups. A *p* value <0.05 was considered to be statistically significant.

## 3. Results

### 3.1. Characterisation of NPs Formulated

**Figure 1:**
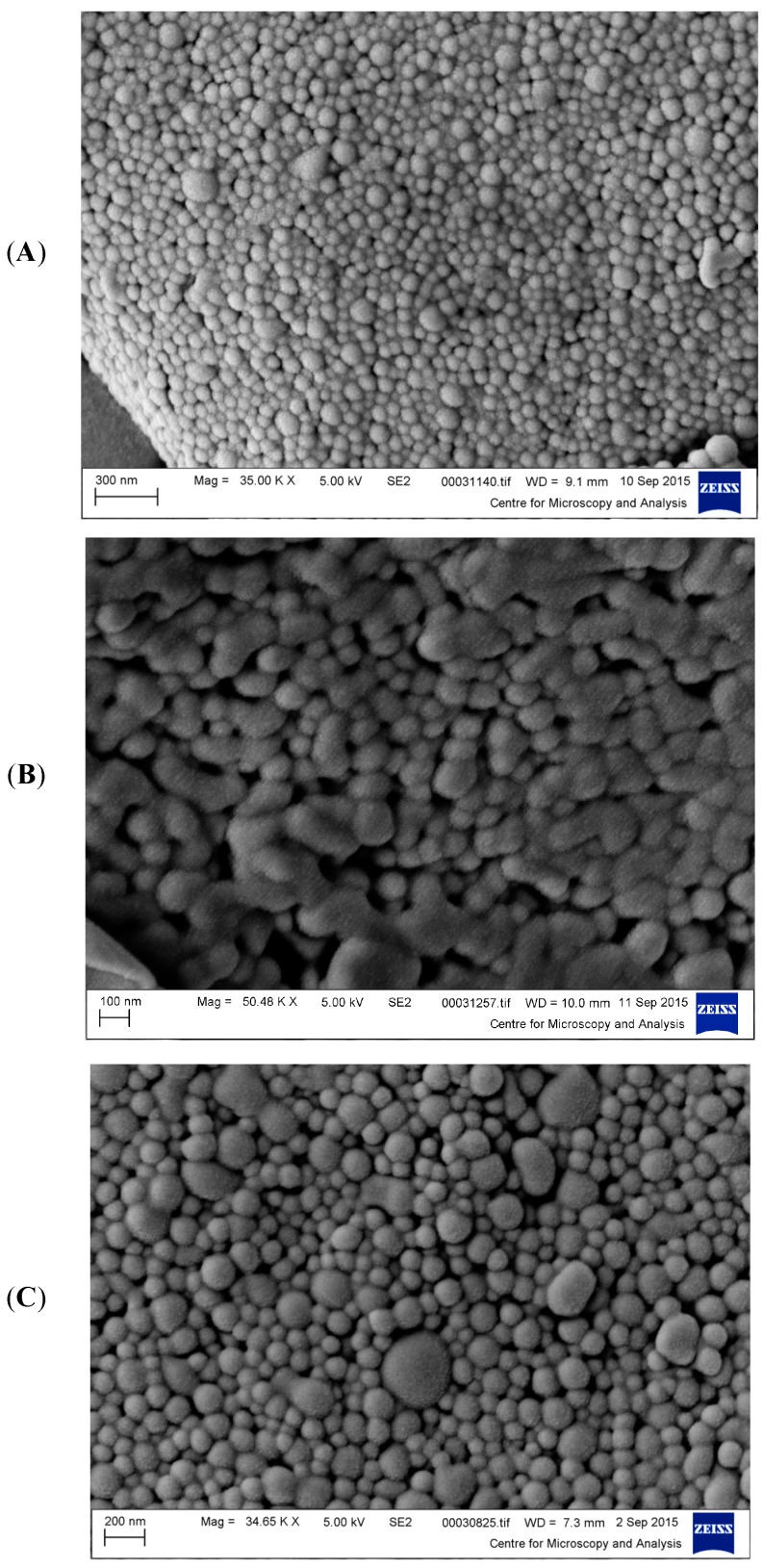
Scanning Electron Microscope Images of PLGA-PEG NPs of average size of (**A**) 112nm, (**B**) 348 nm, and (**C**) 576 nm. Each SEM image is representative of all images taken.

The size, PDI, and zeta potential of the NPs increased with increasing PLGA-PEG concentration (Table 1, Figure 1A–C). A PDI value of 0.10 indicating a nearly monosized distribution was observed for the smallest NPs of 112 nm, while larger PDI values of 0.54 and 0.70 were observed for the 348 and 576 nm NPs, respectively. A larger size distribution is expected for larger size NPs formulated using high polymer concentration. In Figure 1A, the SEM shows the ~100 nm NPs with a distinct spherical shapes and smooth surfaces, exhibiting a narrow range of sizes at different fields of view. In Figure 1B, the NPs of ~350 nm display an interparticular bridging/fusion. In Figure 1C, the NPs of ~600 nm average size maintain a spherical appearance similar to that for the NPs in Figure 1A. The SEMs did not show any internal or external porosity at different magnifications. Calculations by Image J software from SEM analysis showed an average size, which were lower than the sizes measured by DLS. SEM is a tool primarily utilised to analyse particle morphology. The obtained PS determined by microscopy techniques is usually considered the lower limits of PS [25].

The 112 nm NPs had a net negative charge of −22.20 ± 5.71 mV indicating good colloidal stability, whereas the two larger NPs displayed moderately stable colloidal suspensions, with surface charges from −12.40 ± 5.47 to −9.50 ± 14.56 mV. The ZP values of the larger NPs decreased to within the range of −16 to −23 mV on dilution to the concentrations used in the study. The stability of the NPs generated was examined over a 28-day time period to ensure stability over the time period of use. It was noted that storage-induced instability was minimal, particularly in the batches formed of NPs of small and mid-level particle diameters (~100 nm and ~350 nm), with no significant changes in particle properties. Particle PS and PDI properties were retained over this period of 28 days and for the largest size NPs over at least 14 days, revealing a reasonable stability profile for the NPs. This approach in examining the stability of NPs over time is supported by recent studies by Fornaguera and Solans [26].

### 3.2. Effect of Size and Concentration of NPs on Platelet Aggregation

Incubation of NPs with platelets for 4 min in absence of the agonist, thrombin, showed that, irrespective of the size and concentration of NPs (0.05–2.2 mg/mL), spontaneous platelet aggregation was not detected (0 ± 0 % PA, *p* > 0.05, *n* = 3–6). The addition of 0.1 U/mL thrombin, at 4 min in absence of NPs, resulted in a % PA of 72.25–74.75 (Table 2). In presence of 112 nm NPs, no significant difference in % PA was observed after 12 min incubation at all NP concentrations tested (Table 2). Similarly, larger NPs of 348 and 576 nm showed no significant effect on the % PA over the 12 min incubation at the lower NP concentrations of 0.01–1.0 mg/mL and 0.01–1.5 mg/mL for the 348 and 576 nm NPs, respectively (Table 2). At the higher NP concentrations of 1.5 and 2.2 mg/mL, the % PA was significantly lower at 28.40 ± 3.47 and 27 ± 3.10, respectively, when incubated with 348 nm NPs (*p* value < 0.001). Similarly, at the highest concentration of 2.2 mg/mL, NPs of 576 nm resulted in a significantly reduced % PA of 49.00 ± 5.18 (*p* < 0.05) (Table 2).

**Figure 2:**
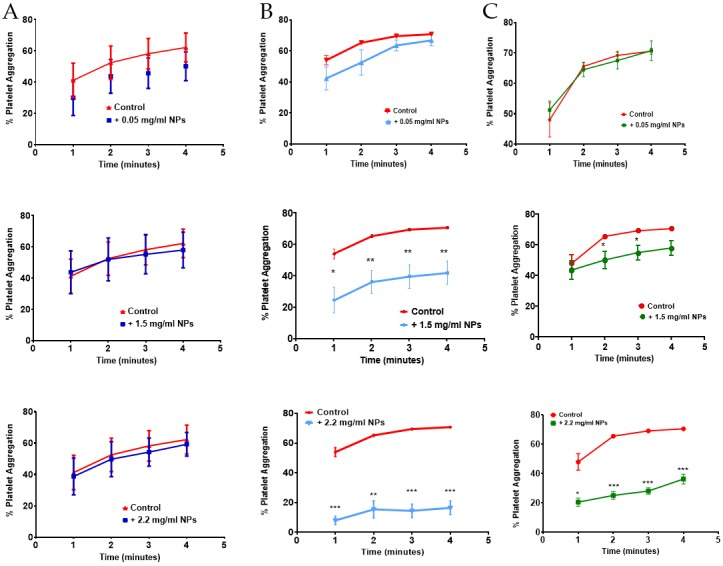
Effect of PLGA-PEG NPs of (**A**) 112 nm, (**B**) 348 nm, and (**C**) 576 nm at 0.05, 1.5, and 2.2 mg/mL on platelet aggregation profile for 4 min following the addition of thrombin. Data are the average of *n* = 4–6 experiments ± SEM. * *p* < 0.05, ** *p* < 0.01, *** *p* < 0.001 compared with 0.1 U/mL thrombin (control), one-way ANOVA, and unpaired student *t*-test, at each time point.

Examination of the platelet aggregation time profile showed no delay in platelet aggregation for the smallest NPs of 112 nm at any of the concentrations tested (Figure 2A). However, the reduced % PA observed at the higher NP concentrations of 348 and 576 nm NPs was associated with a significant delay in platelet aggregation at the earlier time points following addition of thrombin to the sample. Platelet aggregation proceeded significantly more slowly for the 348 and 576 nm NPs at the higher NP concentrations of 1.5 and 2.2 mg/mL (Figure 2B,C), respectively.

### 3.3. Effect of Size of NPs on Platelet Activation Profile

**Figure 3:**
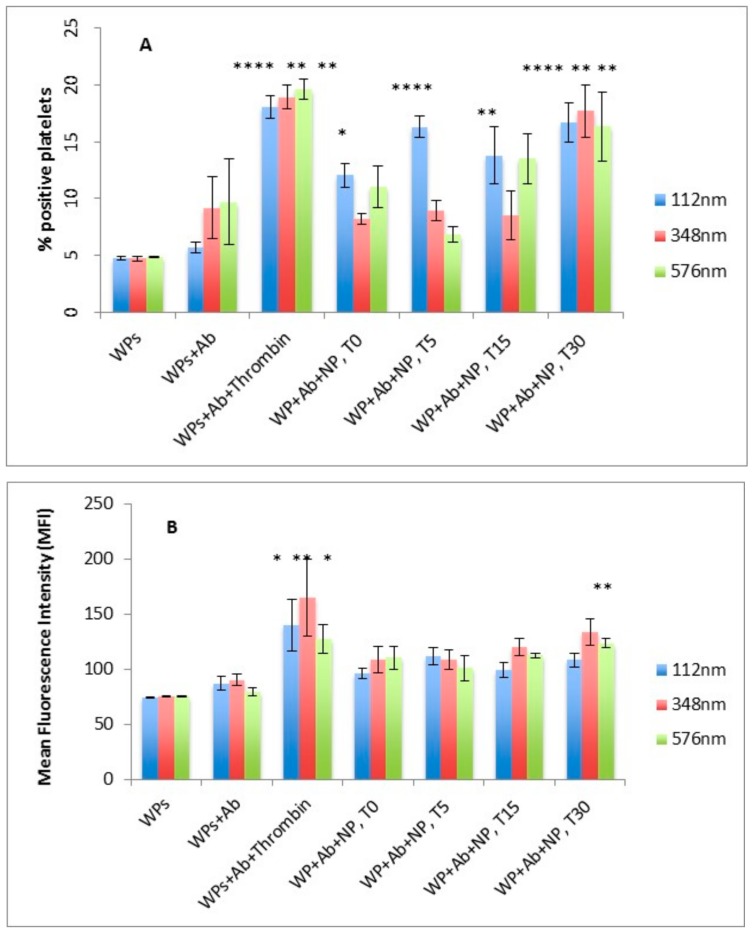
Effect of size of PLGA-PEG NPs at 2.2 mg/mL on platelet activation measured by flow cytometry (**A**) % positive platelets (% PP) (**B**) Mean fluorescence Intensity (MFI). Data are the average of *n* = 3–5 experiments ± SEM. * *p* < 0.05, ** *p* < 0.01, *** *p* < 0.001 compared with washed platelets (WPs) + Ab (control), one-way ANOVA followed by post hoc Dunnett’s test.

The percent positive platelets (% PP) and mean fluorescence intensity (MFI) associated with resting WPs in the absence of NPs were not significantly different when incubated with or without CD62P antibody (Figure 3A,B). On addition of the agonist, thrombin, the % PP and MFI significantly increased as expected, indicating activation of platelets with secretion of CD62P, which binds to the CD62P antibody (*p* < 0.0001 for % PP; *p* < 0.05 for MFI) (Figure 3A,B).

In the presence of PLGA-PEG NPs, a significant increase in % PP for the smallest sized NPs of 112 nm was observed over the 30 min incubation period. However, for the larger NPs of 348 and 576 nm, a significant increase in % PP was only observed at the incubation time of 30 min, (*p* < 0.05, *n* = 3), (Figure 3A). No significant difference in MFI values for platelets incubated with the NPs was observed over the 30 min except for the 348 nm NPs at 30 min incubation (Figure 3B).

### 3.4. Effect of size of NPs on Thrombin Activation of Washed Platelets

**Figure 4:**
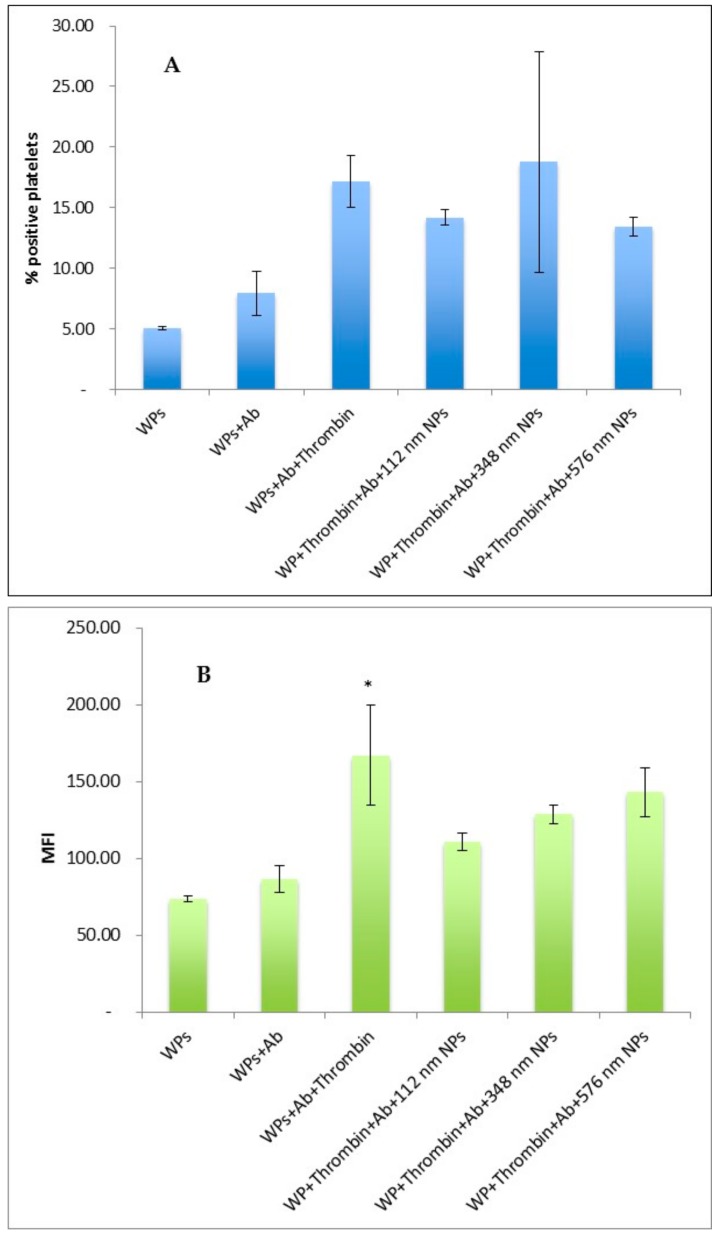
Effect of size of PLGA-PEG NPs at 2.2 mg/mL on thrombin activation of washed platelets measured by flow cytometry (**A**) % positive platelets (% PP) (**B**) Mean fluorescence Intensity (MFI). Data are the average of *n* = 3–5 experiments ± SEM. * *p* < 0.05, compared with WPs + Ab (control), one-way ANOVA followed by post hoc Dunnett’s test.

In absence of NPs, an increase in the percentage of CD6P-positive platelets (% PP) and in CD62P binding level (MFI) were observed following the exposure of WPs to thrombin, as expected, confirming activation of WPs in response to the agonist. The presence of NPs at any of the sizes tested did not result in a significant change in the % PP or MFI following exposure of the WPs with thrombin. (Figure 4A,B).

### 3.5. Confocal Microscopy of the Effect of Incubation Time on the Interaction of NPs and Washed Platelets

**Figure 5:**
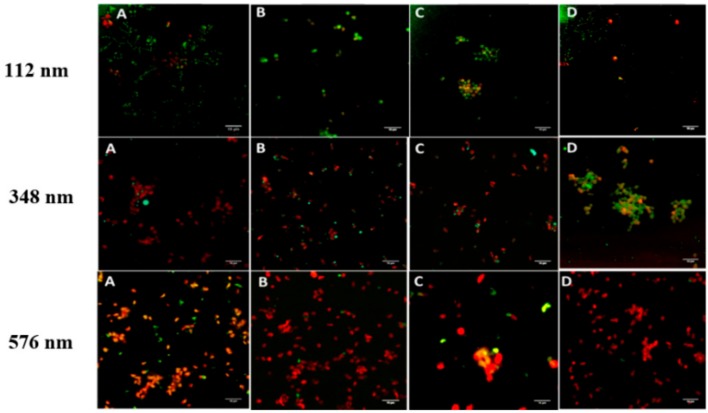
Confocal microscopy images of WPs (red) in suspension incubated with coumarin-PLGA-PEG NPs at 2.2 mg/mL) (**A**) 1 min, (**B**) 5 min, (**C**) 15 min, and (**D**) 30 min, at RT; Magnification 100×. Scale bar represents 10 µm.

A rapid interaction of NPs and WPs, within the first minute, was observed at all NP sizes tested and at both the low (0.01 mg/L) and high (2.2 mg/mL) concentrations of NPs (Figure 5). The percent of platelets bound to NPs of 112 nm was in the order of 44 ± 5.7% and 53.7 ± 7.1% at *t* = 1 min for low and high concentrations, respectively. Increasing the incubation time or concentration of NPs at any of the sizes tested, did not result in significant change in the percent of NPs-bound platelets. The observed interaction between resting platelets and PLGA-PEG NPs of the three sizes tested did not lead to any apparent morphological alteration of the shape of the platelets or development of pseudopods, indicating no effect on platelet activation. This observation was relevant to both low and high concentrations of NPs tested.

**Figure 6:**
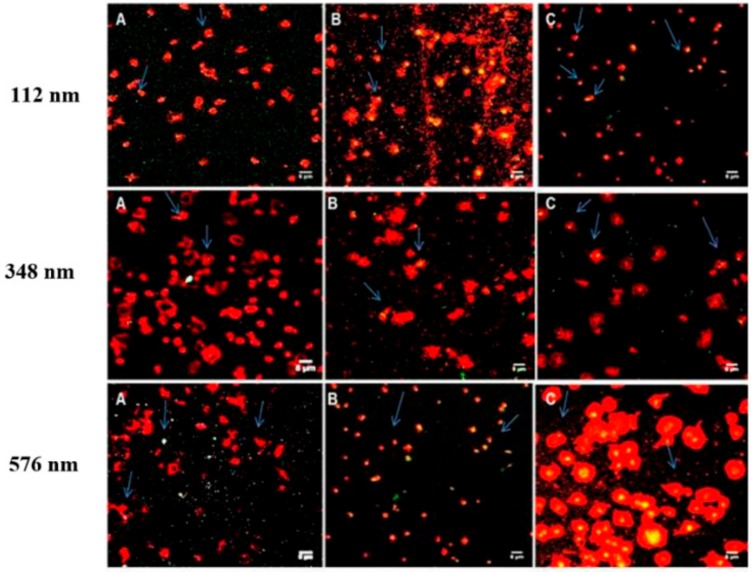
Confocal microscopy images of WPs (red) incubated with coumarin-PLGA-PEG NPs at 2.2 mg/mL and adhered to fibrinogen coated slides: (**A**) 1 min, (**B**) 5 min, and (**C**) 30 min. Magnification 100×. Scale bar represents 10 µm.

Confocal microscopy of platelets layered onto fibrinogen at 20 µg/mL showed the typically formed filopodial projections and heterogeneity expected for adhered platelets. In the presence of NPs, of all of the three sizes and over the incubation times tested, similar attachment of platelets to fibrinogen with alteration in the shape of the platelets and development of extended filopodia projections were observed. The majority of NPs, at all sizes tested, were observed to be bound to platelets (Figure 6). This association of NPs to platelets was of the order of 53.7 ± 1.5% to 59 ± 14.1%, respectively, for 1 and 30 min incubation for 112 nm NPs, at 51 ± 6.1%, and 43 ± 11.3% for 1 and 30 min incubation times, respectively, for the 348 nm NPs. For the 576 nm NP, NP binding was 35.3 ± 5.7% at 1 min and increased to 52.7 ± 2.1% after 30 min incubation. The NP interaction with platelets was maintained upon activation through to adhesion (Figure 6).

## 4. Discussion

The average NP sizes formulated were 112, 348, and 576 nm and were significantly different (*p* < 0.05). This ensured a reasonable comparison of the effects of size of NPs on how platelets may respond when in the presence of NPs with varying sizes. Addition of thrombin at 0.1 U/mL to washed human platelets produced a platelet aggregation of 72–74, which is similar to the percent platelet aggregation of 74 ± 7.8 reported [27]. Incubation of PLGA-PEG NPs, of any size or concentration tested, with WPs for 4 min did not cause any platelet aggregation. This is similar to the results reported for 50 µM silver NPs incubated with WPs for 2 min at 37 °C [22]. In the current study, the 112 nm NPs did not influence the thrombin-induced platelet aggregation profile. Similarly, the larger NPs showed no significant effect on the thrombin-induced platelet aggregation profiles at the lower NP concentrations of 0.01–1.0 mg/mL and 0.01–1.5 mg/mL for the 348 and 576 nm NPs, respectively. Interestingly, a tendency of inhibiting or slowing platelet aggregation was observed for the 348 nm and 576 nm PLGA-PEG NPs at their highest concentrations. This may be related to the presence of NPs in the medium, acting as a physical barrier preventing platelet-platelet contact, hence delaying and reducing platelet activation and aggregation. As the NPs have a negatively charged surface, they may interact with the positively charged thrombin, lowering the free thrombin concentration in the suspension medium, hence reducing the exposure of platelets to thrombin. The surface charge, rather than size, was reported to be the key factor governing this subcategory of NPs’ interaction with platelets [28]. Physiological or non-physiological agents can initiate various signalling pathways in platelets. Platelet activation can also be induced by biomaterials interacting with the specific intrinsic coagulation pathway, which leads to thrombin generation and ultimately platelet activation. However, such mechanisms still remain unexplored [29].

The influence of size-selected NPs on the expression of CD62P on the surface of platelets was analysed by flow cytometry. CD62P is a 140 kD glycoprotein stored in the secretory α-granules of the platelets, mediating leukocyte rolling and platelet–leukocyte aggregation.

The percentage of positive platelets (% PP) for the antibody marker reflects the proportion of activated cells in the total platelet population; this was used to quantify platelet activation in the presence of the NPs. The incubation of the smallest NPs (~113 nm) with WPs resulted in a significant increase in platelet activation, almost similar to the activation observed for thrombin-induced platelet activation. Interestingly, no significant effect on platelet activation was observed in the presence of the larger NPs of 348 nm and the 576 nm. Smaller sized NPs have a higher surface area per unit mass, which result in enhanced interaction potential with platelets. Silica NPs at the smallest PS of 10 nm, at a concentration of 200 µg/mL, were reported to increase CD62P abundance on the platelet surface, while 50 nm-sized NPs did not show any influence [21]. Similarly, of the 10, 50, 113, and 150 nm-sized NPs of silica exposed to platelets, the smallest NPs (10 nm) induced the upregulation of CD62P when used at a low concentration of 10 μg/mL [15,21]. The tendency of 26 nm carboxylated-polystyrene NPs to activate platelets was reported, while particles of larger sizes showed no influence on platelet activation [30].

The presence of NPs of any of the three sizes tested did not influence the % PP or the CD62P abundance on the surface of thrombin-activated platelets. This suggests the investigated NPs did not limit the access of the PE-labelled marker to the WPs surface, irrespective of their sizes.

CLSM performed in this study showed a rapid physical interaction of NPs, of all three sizes examined, with platelets in resting state. This interaction appeared to progress to possible NP uptake by platelets over time. This NP-platelet interaction, however, did not lead to platelet aggregation nor inhibit aggregation of platelets induced by the agonist, thrombin. In contrast, a previous study regarding latex microspheres interaction with platelets suggested platelet activation to be a prerequisite in order to interact with and internalise those inert microparticles (MPs) [31].

The interaction and binding of the investigated NPs to the surface of platelets as well as possible internalisation of NPs into platelets is interesting. In view of their unique small sizes and their curvature, NPs have been shown to enter various human cells and organs, a desirable characteristic for drug targeting [9,24,32].

In the present study, in the presence of PLGA-PEG NPs, variable morphologies of platelets from dendritic spread to the fully spread shape were displayed when platelets were layered onto fibrinogen. This indicates that the NPs did not affect platelet adhesion to fibrinogen. Similarly, silver NPs at different concentrations were reported to have no impact on platelet spreading on fibrinogen [22].

## 5. Conclusions

The results of this study show that while the smallest NPs demonstrated a tendency to result in platelet activation and the larger NPs, at the highest concentrations, to delay platelet aggregation, the three sizes of PLGA-PEG NPs examined had little or no effect on platelet aggregation. The NPs did not interfere with the activation status of platelets and did not inhibit platelet aggregation by the agonist thrombin. Importantly, the rapid binding of the studied PLGA-PEG NPs to platelets is promising, suggesting that these NPs could be potential carriers for targeted drug delivery to platelets in certain disease states. The data presented here supports the use of such NPs as targeting carriers of anti-platelet agents or thrombolytics. It is clear from this study that the question of whether these NPs would be consumed by resting and/or activated platelets remains unanswered. Future strategies may include a more sensitive analysis of the potential for these NPs, pre-loaded with therapeutic drug-candidates, to be internalised by platelets. A number of imaging modalities may be considered such as z stacking, the use of transmission electron microscopy (TEM), or using a combination of high resolution confocal microscopy with flow cytometric analysis that has the potential to provide a precise evaluation of whether NPs are internalised or adsorbed onto the platelets surface, as described by Vranic et al. [33]. With respect to the effects of the interactions of PLGA-PEG NPs at the studied sizes on normal platelet functions, the use of a more sensitive platelet aggregation assay, such as flow-induced platelet aggregation in the presence of PLGA-PEG NPs, should be considered in future studies. An example would include a flow-mimicking technique such as quartz crystal microbalance with dissipation (QCM-D), which operates under similar flow conditions encountered in microvasculature [34]. Further understanding of the interaction between platelets and NPs is required to aid in the development of a safe platelet-targeted drug delivery system in the future.

## Figures and Tables

**Table 1:** Physicochemical characteristics of nanoparticles (NPs) used in platelet aggregation studies. *n* = 5 batches ± SD. PLGA = poly-lactide-*co*-glycolide; PEG = poly ethylene glycol.

PLGA-PEG (mg/mL)	PS (nm)	PDI	ZP (mV)
10	111.55 ± 5.81	0.10 ± 0.02	−22.20 ± 5.71
55	348.00 ± 23.61	0.54 ± 0.04	−12.40 ± 6.30
100	576.19 ± 6.82	0.70 ± 0.06	−9.50 ± 14.56

PLGA: poly-lactide-*co*-glycolide; PEG: polyethylene glycol; PS: Particle size; PDI: Polydispersity Index, ZP: Zeta Potential.

**Table 2:** Effect of size and concentration of PLGA-PEG NPs on percent platelet aggregation (% PA). Data are the average of *n* = 4–6 experiments ± SEM. * *p* < 0.05, ** *p* < 0.01, *** *p* < 0.001 compared with 0.1 U/mL thrombin (control), one-way ANOVA, and unpaired student *t*-test, at each time point.

PLGA-PEG NPs (mg/mL)	% PA (112 nm)	% PA (348 nm)	% PA (576 nm)
0 (Control)	72.75 ± 1.89	74.75 ± 1.93	72.25 ± 0.62
0.05	72.00 ± 0.91	73.50 ± 1.88	75.00 ± 4.63
0.1	73.00 ± 1.23	76.20 ± 2.99	69.75 ± 8.22
0.25	69.00 ± 1.68	66.00 ± 5.11	74.00 ± 4.89
0.5	73.50 ± 0.96	70.25 ± 4.13	68.00 ± 4.97
1	72.75 ± 0.85	62.50 ± 3.59	71.25 ± 5.27
1.5	72.25 ± 1.55	28.40 ± 3.47***	68.75 ± 2.10
2.2	71.00 ± 1.80	27.00 ± 3.10***	49.00 ± 5.18*
